# Changes in the expression of GABA_A_ receptor subunit mRNAs in parahippocampal areas after kainic acid induced seizures

**DOI:** 10.3389/fncir.2013.00142

**Published:** 2013-09-18

**Authors:** Meinrad Drexel, Elke Kirchmair, Günther Sperk

**Affiliations:** Department of Pharmacology, Innsbruck Medical UniversityInnsbruck, Austria

**Keywords:** epilepsy, tonic inhibition, GABA_A_-receptor, temporal lobe epilepsy, subiculum, entorhinal cortex, epileptogenesis

## Abstract

The parahippocampal areas including the subiculum, pre- and parasubiculum, and notably the entorhinal cortex (EC) are intimately involved in the generation of limbic seizures in temporal lobe epilepsy. We investigated changes in the expression of 10 major GABA_A_ receptor subunit mRNAs in subfields of the ventral hippocampus, ventral subiculum, EC, and perirhinal cortex (PRC) at different intervals (1, 8, 30, and 90 days) after kainic acid (KA)-induced status epilepticus priming epileptogenesis in the rat. The most pronounced and ubiquitous changes were a transient (24 h after KA only) down-regulation of γ2 mRNA and lasting decreases in subunit α5, β3, and δ mRNAs that were prominent in all hippocampal and parahippocampal areas. In the subiculum similarly as in sectors CA1 and CA3, levels of subunit α1, α2, α4, and γ2 mRNAs decreased transiently (1 day after KA-induced status epilepticus). They were followed by increased expression of subunit α1 and α3 mRNAs in the dentate gyrus (DG) and sectors CA1 and CA3, and subunit α1 also in the EC layer II (30 and 90 days after KA). We also observed sustained overexpression of subunits α4 and γ2 in the subiculum and in the Ammon’s horn. Subunit γ2 mRNA was also increased in sector CA1 at the late intervals after KA. Taken together, our results suggest distinct regulation of mRNA expression for individual GABA_A_ receptor subunits. Especially striking was the wide-spread down-regulation of the often peri- or extrasynaptically located subunits α5 and δ. These subunits are often associated with tonic inhibition. Their decrease could be related to decreased tonic inhibition or may merely reflect compensatory changes. In contrast, expression of subunit α4 that may also mediate tonic inhibition when associated with the δ-subunit was significantly upregulated in the DG and in the proximal subiculum at late intervals. Thus, concomitant up-regulation of subunit γ2, α1 and α4 mRNAs (and loss in δ-subunits) ultimately indicates significant rearrangement of GABA_A_ receptor composition after KA-induced seizures.

## INTRODUCTION

Temporal lobe epilepsy (TLE) is the most common and difficult to treat form of focal epilepsies. It comprises about 30% of all epilepsies (Fisher et al., 1998). The most common pathology underlying TLE is hippocampal damage, termed Ammon’s horn sclerosis, and primarily affects the hilus of the dentate gyrus (DG) and hippocampal sectors CA3 and CA1 while other brain areas are considerably less affected (Babb et al., 1984). In recent years, however, neurodegeneration and epilepsy-induced neurochemical changes were found also in areas closely associated with the hippocampus, such as the subiculum (Andrioli et al., 2007) and the entorhinal cortex (EC; Du et al., 1993; Bartolomei et al., 2005). These brain regions may also be intimately involved in seizure propagation in human TLE (Cohen et al., 2002; Wozny et al., 2005; Fabo et al., 2008; Huberfeld et al., 2011) and in animal models of TLE (Knopp et al., 2005; de Guzman et al., 2006; Kumar and Buckmaster, 2006). Malfunctioning of GABAergic transmission is one of the major hypotheses for generation of epilepsy. Thus, preferential losses of GABAergic neurons have been proposed to be responsible for impaired inhibition (Houser et al., 1986; Sloviter, 1987; Andre et al., 2001; Dinocourt et al., 2003). Reports showing overexpression of neurochemical markers, such as glutamate decarboxylases or neuropeptides, indicating *enhanced* GABAergic transmission in surviving GABA neurons, however challenged this view (Marksteiner and Sperk, 1988; Esclapez and Houser, 1999; Sperk et al., 2003). Several groups, however, demonstrated a selective loss of parvalbumin-containing interneurons (Best et al., 1993; DeFelipe et al., 1993; Knopp et al., 2008; Drexel et al., 2011) or down-regulation of parvalbumin (Wittner et al., 2001; Magloczky and Freund, 2005) in these neurons in the sector CA1 and the subiculum of epileptic rats and in TLE patients. Also a role of possibly impaired GABAergic transmission through altered GABA_A_ or GABA_B_ receptors has been extensively investigated (Kamphuis et al., 1995; Sperk et al., 1998; Furtinger et al., 2003). One of the initially unexpected findings was that GABA_A_ receptor binding is *increased*, not decreased in kindled rats (Shin et al., 1985). Later an altered subunit constitution of GABA_A_ receptors and consequently altered GABAergic transmission was proposed as a cause of epileptogenesis (Sperk et al., 1998, 2004). Several groups performed neurochemical and electrophysiological experiments in rat models and in hippocampal tissue removed from patients suffering from TLE (Loup et al., 2000; Pirker et al., 2003; Peng et al., 2004; Nishimura et al., 2005). These studies mainly focused on the hippocampal formation and epilepsy-induced changes included increased expression of α4-, γ2-, and β-subunits going along with decreased expression of δ-subunit in the DG or down-regulation of α5-subunits in CA1 pyramidal cells (Schwarzer et al., 1997; Tsunashima et al., 1997; Loup et al., 2000; Houser and Esclapez, 2003; Peng et al., 2004). The changes observed indicate distinct effects of epilepsy on subunits implicated in phasic or tonic GABAergic neurotransmission, respectively.

Most studies in animal models of TLE so far focused on changes in GABA_A_ receptor subunit expression in the dorsal hippocampus including the DG and the Ammon’s horn (Sperk et al., 1998, 2004). We recently became aware of a crucial role of parahippocampal areas, notably of the subiculum and the EC in epileptogenesis. Recent key findings were increased excitability of the subiculum in rodent models of TLE (Knopp et al., 2005; de Guzman et al., 2006) and in tissue obtained from TLE surgery (Cohen et al., 2002; Wozny et al., 2003; Huberfeld et al., 2011), massive losses of parvalbumin-expressing interneurons in the subiculum and in deep layers of the EC (Andrioli et al., 2007; Knopp et al., 2008; Drexel et al., 2011), and a correlation of the loss of parvalbumin-expressing interneurons in the subiculum with the numbers of spontaneous seizures in the rat kainic acid (KA)-model of TLE (Drexel et al., 2011).

To elucidate possible changes in GABAergic transmission in parahippocampal areas we investigated changes in GABA_A_ receptor subunit expression in the ventral hippocampus including the subiculum and the entorhinal and perirhinal cortices.

## MATERIALS AND METHODS

### ANIMALS

Adult male Sprague-Dawley rats (220–250 g; Institut für Versuchstierzucht, Himberg, Austria) were used in the study. The rats were housed in single-ventilated cages at a temperature of 22–24°C, a relative humidity of 50–60%, and a 12 h light/dark cycle. They had access to food and water *ad libitum*. All animal experiments were conducted according to national guidelines and European Community laws and were approved by the Committee for Animal Protection of the Austrian Ministry of Science.

### KAINIC ACID INJECTION

Twenty-nine rats were injected i.p. with 10 mg/kg KA (5 mg/ml in saline, pH 7.0, Ascent Scientific, Bristol, UK) and 13 control rats with saline. Two hours after the first generalized seizure the rats were treated with diazepam (10 mg/kg, i.p., Gewacalm, Nycomed Austria GmbH, Linz, Austria) to reduce mortality and severity of the neuropathological outcome. Their seizure behavior was investigated for at least 3 h and rated according to a five-stage rating scale described previously (Sperk et al., 1983). Rats without obvious behavioral changes were rated as stage 0, rats showing wet dog shakes only as stage 1, rats with chewing, head bobbing and forelimb cloni as stage 2, rats with generalized seizures and rearing as stage 3, rats with generalized seizures, rearing and loss of postural tone (falling over) as stage 4, and rats that died during status epilepticus were rated as stage 5. Only rats exhibiting rating 3 or 4 were used. In brains of 19 rats (+9 controls) *in situ* hybridization and in four rats (+4 controls) neuron specific nuclear protein (NeuN) immunohistochemistry was performed 30 days after injection of KA.

### TISSUE PREPARATION

For *in situ* hybridization, rats were killed by exposure to CO_2_-gas either 1 day (*n* = 5), 8 days (*n* = 6), 30 days (*n* = 5), or 90 days (*n* = 3) after KA-induced status epilepticus. Controls were killed 1 day (*n* = 3), 30 days (*n* = 3), or 90 days (*n* = 3) after saline injection. These intervals were chosen for assessing changes directly related to consequences of the status epilepticus (1 day), to changes in the presumed silent phase (8 days), and changes due to the chronic epilepsy syndrome (30 and 90 days). Brains were quickly removed and snap-frozen in isopentane (-70°C). Horizontal 20 μm sections were cut using a cryostat-microtome (Microm HM 560 M, Carl Zeiss AG, Vienna, Austria), thaw-mounted on silane-coated slides and stored at -70°C. Every 11th section was stained with cresyl violet, dehydrated, cleared in butyl acetate, and coverslipped using *Eukitt* mounting medium (O. Kindler GmbH, Freiburg, Germany). These sections were used for matching the individual brains at the same anatomical level along the dorso-ventral axis for later histochemistry.

### *IN SITU* HYBRIDIZATION

*In situ* hybridization was performed as described previously in detail (Tsunashima et al., 1997). The sequences of custom-synthesized oligonucleotides (Microsynth AG, Balgach, Switzerland) complementary to the respective mRNAs for GABA_A_ receptor subunits that were used as probes are listed in **Table 1**.

**Table 1:** Oligonucleotide sequences used for *in situ* hybridization.

mRNA	Access code	Oligonucleotide sequence
α1	NM_010250.4	5′ CCT GGC TAA GTT AGG GGT ATA GCT GGT TGC TGT AGG AGC ATA TGT 3′
α2	NM_001135779.1	5′ AGG ATC TTT GGA AAG ATT CGG GGC GTA GTT GGC AAC GGC TAC AGC 3′
α3	NM_017069.2	5′ ATA GGT GGT TCC CAC TAT GTT GAA GGT GGT GCT TGT TTT CTT GGT 3′
α4	NM_080587.3	5′ CAA GTC GCC AGG CAC AGG ACG TGC AGG AGG GCG AGG CTG ACC CCG 3′
α5	NM_017295.1	5′ TTC CCA GTC CCG CCT GGA AGC TGC TCC TTT GGG ATG TTT GGA GGA 3′
β1	NM_012956.1	5′ TGC CTG TCC AGC CCT CGT CCG AAG CCC TCA CGG CTG CTC AGT GGT 3′
β2	X_15467.1	5′ ACT GTT TGA AGA GGA ATC TAG TCC TTG CTT CTC ATG GGA GGC TGG 3′
β3	NM_008071.3	5′ CTG TCT CCC ATG TAC CGC CCA TGC CCT TCC TTG GGC ATG CTC TGT 3′
γ2	NM_183327.1	5′ GCG AAT GTG TAT CCT CCC GTG TCT CCA GGC TCC TGT TCG G 3′
δ	NM_017289.1	5′ GGT CCA TGT CAC AGG CCA CTG TGG AGG TGA TGC GGA TGC T 3′

Briefly, the oligonucleotides (2.5 pmol) were labeled at the 3′-end with [^35^S] α-thio-dATP (1,300 Ci/mmol; New England Nuclear, Boston, MA, USA) by reaction with terminal deoxynucleotidyltransferase (Roche Austria GmbH, Vienna, Austria) and precipitated with 75% ethanol and 0.4% NaCl. Frozen sections (20 μm) were immersed in ice-cold paraformaldehyde (2%) in phosphate-buffered saline (PBS), pH 7.2 for 10 min, rinsed in PBS, immersed in acetic anhydride (0.25% in 0.1 mol/l triethylamine hydrochloride) at room temperature for 10 min, dehydrated by ethanol series, and delipidated with chloroform. The sections were then hybridized in 50 μl hybridization buffer containing about 50 fmol (0.8 to 1 × 10^6^ cpm) labeled oligonucleotide probe for 18 h at 42°C. The hybridization buffer consisted of 50% formamide (Merck, Darmstadt, Germany), 2× SSC (1× SSC consisting of 150 mmol/l NaCl and 15 mmol/l sodium citrate, pH 7.2). The sections were then washed twice in 50% formamide in 1 × SSC (42°C, 4 × 15 min), briefly rinsed in 1 × SSC, rinsed in water, dipped in 70% ethanol, dried, and then exposed to BioMax MR films (Sigma-Aldrich, Vienna, Austria) together with [^14^C]-microscales for 7–42 days. After exposure to BioMax MR films, the sections were dipped at 42°C in photosensitive emulsion (NTB-2; Kodak, Rochester, NY, USA) diluted 1:1 with distilled water, air dried, and exposed for 14–40 days. Dipped sections and BioMax films were developed using Kodak D19 developer (Sigma-Aldrich, Vienna, Austria). After counterstaining with cresyl violet, photoemulsion-dipped sections were dehydrated, cleared in butyl acetate, and coverslipped with *Eukitt*.

### DENSITOMETRICAL ANALYSIS OF mRNA EXPRESSION

Autoradiographic films were digitized and opened in NIH ImageJ (version 1.46; U.S. National Institutes of Health, Bethesda, ML, USA; ). The following regions were investigated for epilepsy-induced changes in mRNA expression of individual GABA_A_ receptor subunits: the granule cell layer of DG, pyramidal cell layers of hippocampal sectors CA3, CA1, and the proximal and distal parts of the subiculum, layers II and V/VI of the medial and lateral EC and layers II/III of the PRC. As the values obtained in the medial and lateral EC were not significantly different from each other, they were pooled. Briefly, a line selection (20 pixels width) was drawn perpendicular to the layer of interest and a density profile plot (gray values) was created using the function “analyze – plot profile.” Values for relative optical densities (RODs) were calculated from gray values according to the following formula: ROD = log[256/(255 - gray value)]. ROD values obtained from left and right hemispheres were averaged and film background ROD was subtracted. By comparing the measures with those obtained with autoradiography standards we took care to take our ROD measures strictly in a linear range. In cases where this was not the case we reduced the autoradiographic exposure times.

### IMMUNOHISTOCHEMISTRY FOR NeuN

For immunohistochemistry, additional KA-injected rats (*n* = 4, rating 3–4) and saline-injected controls (*n* = 4) were transcardially perfused with 4% paraformaldehyde (Merck, Darmstadt, Germany) 30 days after the treatment and subjected to peroxidase–antiperoxidase immunohistochemistry for NeuN as described before (Drexel et al., 2011).

### STATISTICAL ANALYSIS OF *IN SITU* HYBRIDIZATION DATA

Statistical analysis was carried out using GraphPad Prism 5.0a for Macintosh (GraphPad Software, San Diego, CA, USA). Analysis of variance (ANOVA) with Dunnett’s multiple comparison *post hoc* test was used for determining between-group differences among multiple sets of data. All data are presented as mean ± SEM. Statistical significance was defined as *p* < 0.05.

## RESULTS

### BEHAVIORAL CHANGES

Among the 29 rats injected with KA, 23 rats developed stage 3–4 seizures. One and two rats revealed stage 2 and 1 seizures, respectively, and three rats died during status epilepticus. These behavioral responses to KA injection were highly comparable to our previous data (Drexel et al., 2012). Only rats with stage 3–4 seizures were included in the study. In our present experiment we did not perform EEG recordings (Drexel et al., 2012). In our recent experiments, however, we observed spontaneous EEG seizures (1.4 per day) in all rats that had responded with an acute status epilepticus upon i.p. KA injection (in the same way as described here). The duration of the silent period was variable in these experiments and lasted between 3 and 36 days (mean: 14.9 ± 1.43 days; Drexel et al., 2012).

### HISTOPATHOLOGY

**FIGURE 1:**
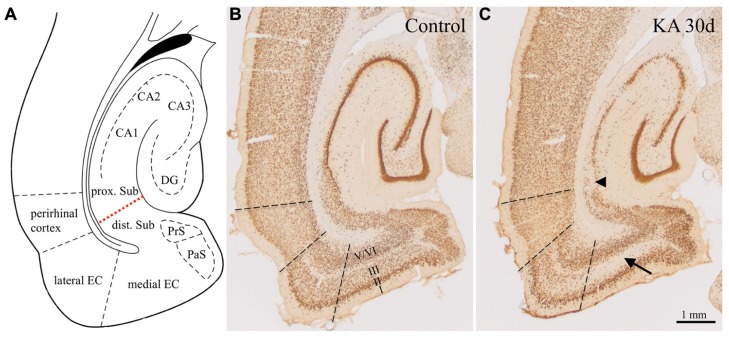
**Epilepsy-induced neurodegeneration in the ventral hippocampal/parahippocampal formation of the rat (neuron specific nuclear protein, NeuN-immunoreactivity).** **(A)** Anatomical overview of the subregions of the ventral hippocampal/parahippocampal formation. **(B)** Horizontal brain section of a control rat labeled for NeuN (Bregma -6.1 mm). **(C)** NeuN-labeling 30 days after KA-induced status epilepticus. Cell losses were most prominent in layer III of the medial EC (arrow), in the proximal part of the subiculum (arrowhead) and in the pyramidal cell layer of the hippocampus proper. DG, dentate gyrus; EC, entorhinal cortex; PrS, presubiculum; PaS, parasubiculum; dist. and prox. Sub, distal and proximal subiculum; CA1–CA3, hippocampal sectors CA1 and CA3. Losses in NeuN-positive neurons were up to 60% in layer III of the medial EC, up to 45% in the proximal subiculum, up to maximal 25% in the distal subiculum and pre- and parasubiculum, and layers II and V/VI of the EC Drexel et al., 2012).

Apart from neuronal losses in the hilus of the DG and degeneration of CA3- and CA1-pyramidal neurons the rats displayed widespread losses of principal neurons and GABAergic interneurons in the subiculum and in subareas of the parahippocampal region (**Figure 1**). As shown previously, cell losses occurred already 1 day after KA-induced status epilepticus and were most intense in layer III of the medial EC (about -50%; **Figure 1C**, arrow) and in the proximal subiculum (about -40%; **Figure 1C**, arrowhead; Drexel et al., 2012).

### DISTRIBUTION OF GABA_A_ RECEPTOR SUBUNIT mRNAs IN CONTROLS

#### Distribution of subunit α1–α5 mRNAs

**FIGURE 2:**
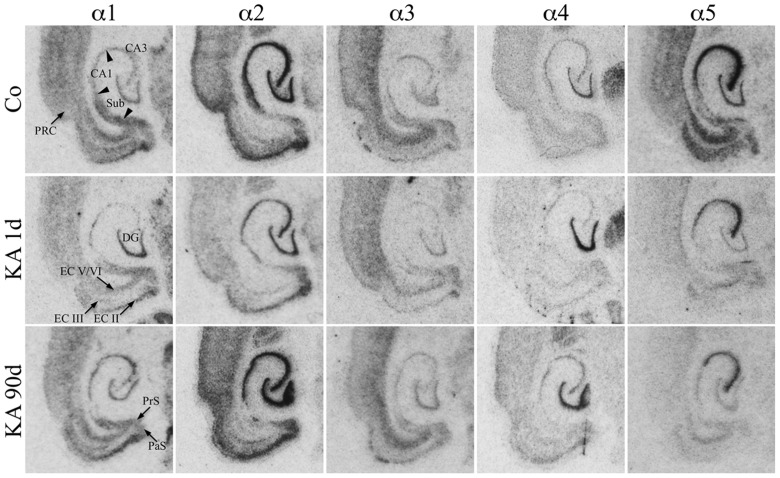
**Expression of α-subunit mRNAs after KA-induced seizures.** Autoradiographs of horizontal sections of the hippocampus and parahippocampal areas after *in situ* hybridization for GABA_A_ receptor subunits α1–α5 in untreated controls (Co) and at different intervals after KA-induced status epilepticus are depicted. Subunit α1–α5 mRNAs were expressed in all principal cell layers of the hippocampal/parahippocampal formation of controls. Note the sustained down-regulation of α5 mRNA throughout the hippocampal formation. On the other hand, subunits α1 (CA3, subiculum, EC, PRC) and α2 (in most regions) were only transiently reduced 1 day after KA-induced seizures. Subunit α3 mRNA is only moderately altered, whereas subunit α4 mRNA is upregulated in the dentate gyrus at all intervals.

For the hippocampus proper and the DG the subunit distribution was rather similar as that described for the dorsal and ventral hippocampus of the rat (Wisden et al., 1992; Tsunashima et al., 1997) and for the ventral hippocampus of the mouse (Hortnagl et al., 2013). To our knowledge no comprehensive study on the expression of all GABA_A_ receptor subunits in horizontal sections of parahippocampal areas of the rat is yet available. As shown in **Figure 2**, mRNAs for the α-subunits were abundantly expressed in all principal cell layers of the ventral hippocampal formation (DG, hippocampus proper, and subiculum) and parahippocampal region (presubiculum, parasubiculum, EC, and PRC) of saline-injected rats.

Transcripts for α2 and α5 were especially abundant. Strongest expression of α2 mRNA was present in the granule cell layer of the DG, in the pyramidal cell layer of the hippocampus proper, and in the subiculum as well as in layer II of the EC and throughout the PRC (**Figure 2**). Expression of subunit α5 was strongest in sectors CA1–CA3 and in the EC and somewhat less prominent in the stratum granulosum. In the PRC, it was predominantly expressed in the deepest layers. It was weaker in the proximal subiculum and in the presubiculum.

Subunit α1 was almost equally distributed throughout the granule cell layer and the stratum pyramidale CA1–CA3. It was even more prominent in the subiculum, pre-, and parasubiculum and in the EC notably in layers II/III and in deep layers. Its presence in the hilus of the DG indicates expression in hilar interneurons. Subunit α3 expression appeared to be weaker (**Figure 2**). It was strongest in the deep layers of the EC and PRC and more prominent in sector CA3 than in sector CA1 and in the stratum granulosum. Interestingly, clear labeling of the hilus of the DG was observed presumably reflecting the location of the α3-subunit on hilar interneurons. Subunit α4 mRNA was concentrated in the granule cell layer of the DG while the remaining subregions revealed only modest expression levels.

#### Distribution of subunit β1–β3 mRNAs

**FIGURE 3:**
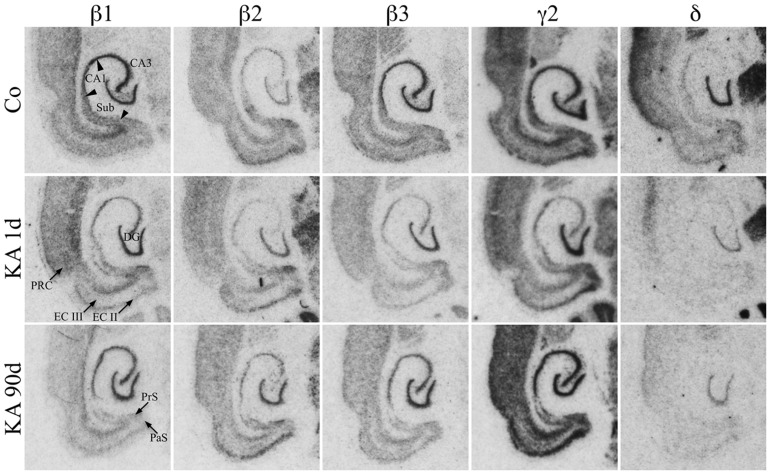
**Expression of subunit β1, β2, β3, γ2, and δ mRNAs after KA-induced seizures.** Autoradiographs of horizontal sections of the hippocampus and parahippocampal areas after *in situ* hybridization for GABA_A_ receptor subunits β1, β2, β3, γ2, and δ in saline-injected controls (Co) and at different intervals after KA-induced status epilepticus are shown. Messenger RNAs encoding for β-subunits and for δ appear to be widely reduced, those for γ2 appear to increase after an initial (1 day) reduction.

As shown in **Figure 3**, mRNAs for all three β-subunits were distributed throughout principal cell layers of all hippocampal and parahippocampal areas including the PRC. For β1 and β3 it was somewhat more prominent in the granule and pyramidal cell layers than in parahippocampal areas. Subunit β2 mRNA appeared to be slightly more concentrated in the EC and in the DG than in hippocampal pyramidal cells and showed a somewhat weaker expression in the subiculum and PRC (**Figure 3**). All three subunits were also expressed in interneurons of the dentate hilus (**Figure 3**).

#### Distribution of subunit γ2 and δ mRNAs

Subunit γ2 mRNA was strongly expressed in all principal cell layers of the hippocampal formation and parahippocampal regions including the PRC (**Figure 3**). In the EC especially layers II and V/VI showed prominent γ2 mRNA expression. Labeling of the dentate hilus indicates expression of the γ2 subunit in hilar interneurons. Subunit δ mRNA was highly expressed in the dentate granule cell layer and in the superficial layers of the PRC but not or only very weakly in all other hippocampal and parahippocampal regions (**Figure 3**).

### CHANGES IN THE EXPRESSION OF GABA_A_ RECEPTOR SUBUNITS AFTER KA-INDUCED STATUS EPILEPTICUS

**FIGURE 4:**
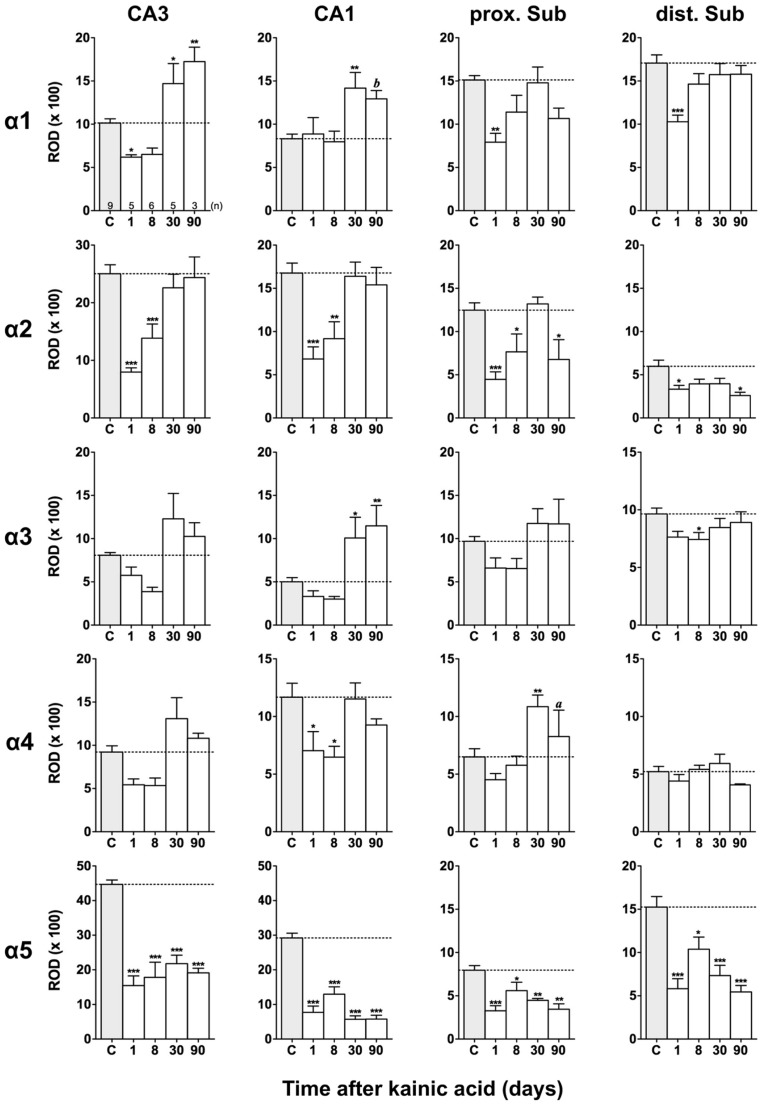
**Semi-quantitative assessment of mRNA levels for GABA_**A**_ receptor subunits α1–α5 in the hippocampus proper and subiculum after KA-induced seizures.** The autoradiographic films were digitized and analyzed using the open source NIH ImageJ software. Note the lasting down-regulation of subunit α5 mRNA levels in the hippocampus proper and subiculum, whereas mRNAs of the other α subunits are only transiently down-regulated. α2 mRNA levels are still reduced in the subiculum after 90 days. Data are expressed as mean relative optical densities (RODs) ± SEM. Numbers of animals are given in the upper left graph. Statistical analysis was done by ANOVA and Dunnett’s multiple comparison *post hoc* test (*p < 0.05; **p < 0.01; ***p < 0.001). In cases where, due to the low number of animals, no significant difference was found for the 30 and 90 days intervals, data for this time points were pooled and re-analyzed: *a*, *p* < 0.05; *b*, *p* < 0.01.

**FIGURE 5:**
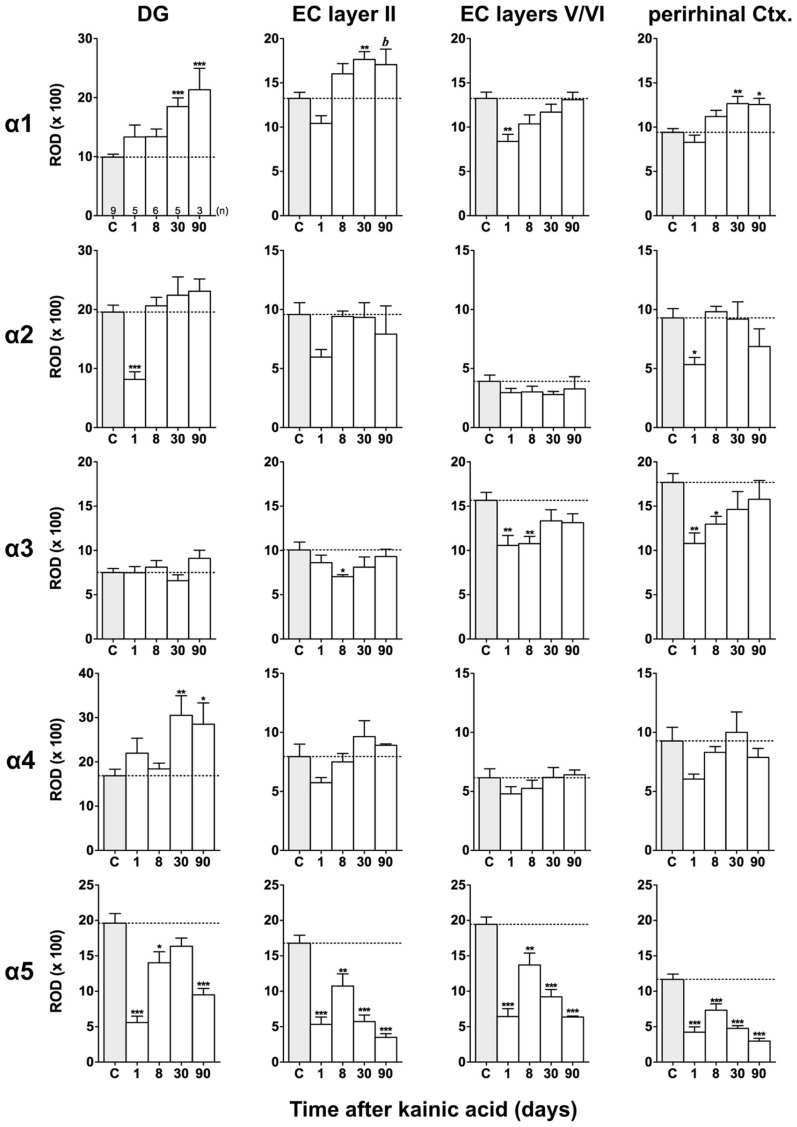
**Semi-quantitative assessment of mRNA levels for GABA_**A**_ receptor subunits α1–α5 in the dentate gyrus (DG), entorhinal cortex (EC), and perirhinal cortex (PRC) after KA-induced seizures.** Density profile plots were performed in layers of the hippocampal/parahippocampal region on autoradiograms after radioactive *in situ* hybridization and relative optical densities (RODs) were calculated. Note the lasting down-regulation of subunit α5 mRNA in the DG, EC, and PRC and concomitant up-regulation of subunit α1 mRNA (DG, EC layer II, PRC) and α4 mRNA (onlyDG) at late intervals after status epilepticus. Data are given as mean ROD values ± SEM. Animal numbers are given in the upper left graph. Statistical analysis was done by ANOVA and Dunnett’s multiple comparison *post hoc* test (*p < 0.05; **p < 0.01; ***p < 0.001; 30 and 90 days pooled: *b*, *p* < 0.01).

**FIGURE 6:**
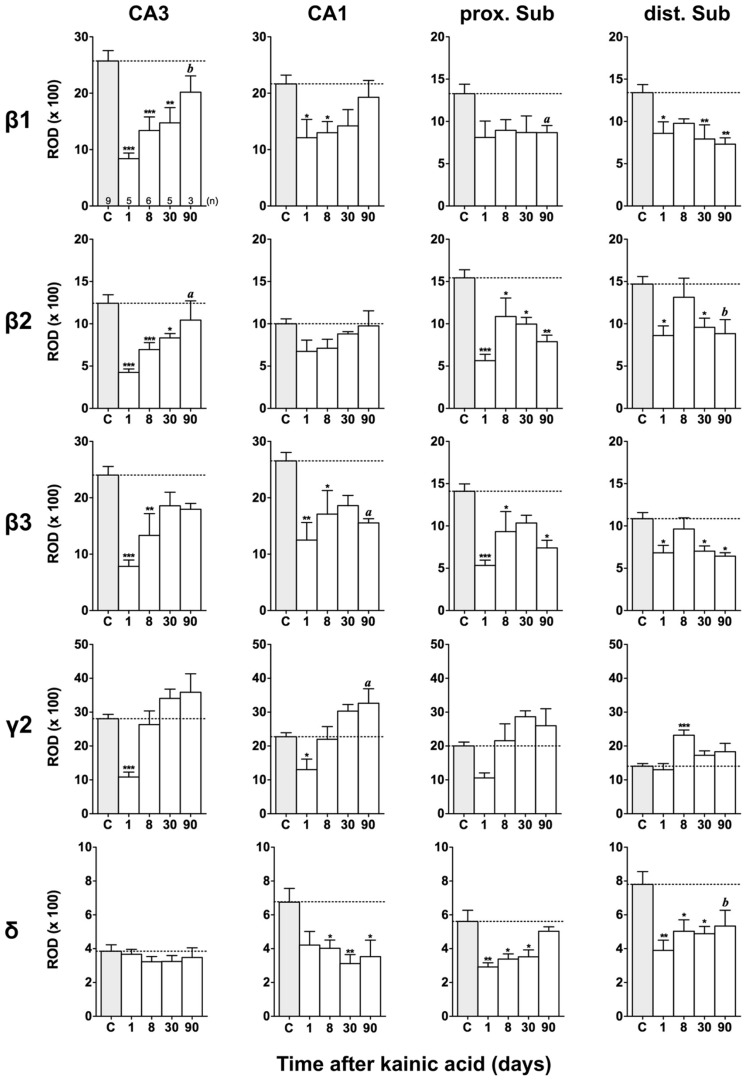
**Semi-quantitative assessment of mRNA levels for GABA_**A**_ receptor subunits β1–β3, γ2, and δ in the hippocampus proper and subiculum at different time intervals after KA-induced seizures.** Note reduced β1-β3 mRNA levels in the hippocampus proper and subiculum 24 h after KA increasing again in the hippocampus proper at later intervals. In the subiculum (β1-β3) levels remain reduced 90 days after KA. Subunit mRNA δ2 levels are transiently decreased in the hippocampus proper and proximal subiculum 1 day after status epilepticus, however, increasing at later intervals. Levels of the δ-subunit are permanently reduced in sector CA1 and in the distal subiculum. Numbers of rats per group are given in the upper left graph. Data are expressed as mean ROD ± SEM; statistical analysis was done by ANOVA and Dunnett’s multiple comparison *post hoc* test (*p < 0.05; **p < 0.01; ***p < 0.001). In cases where, due to the low number of animals, no significant difference was found for the 30 and 90 days intervals, data for this time points were pooled and re-analyzed: a, *p* < 0.05; b, *p* < 0.01.

**FIGURE 7:**
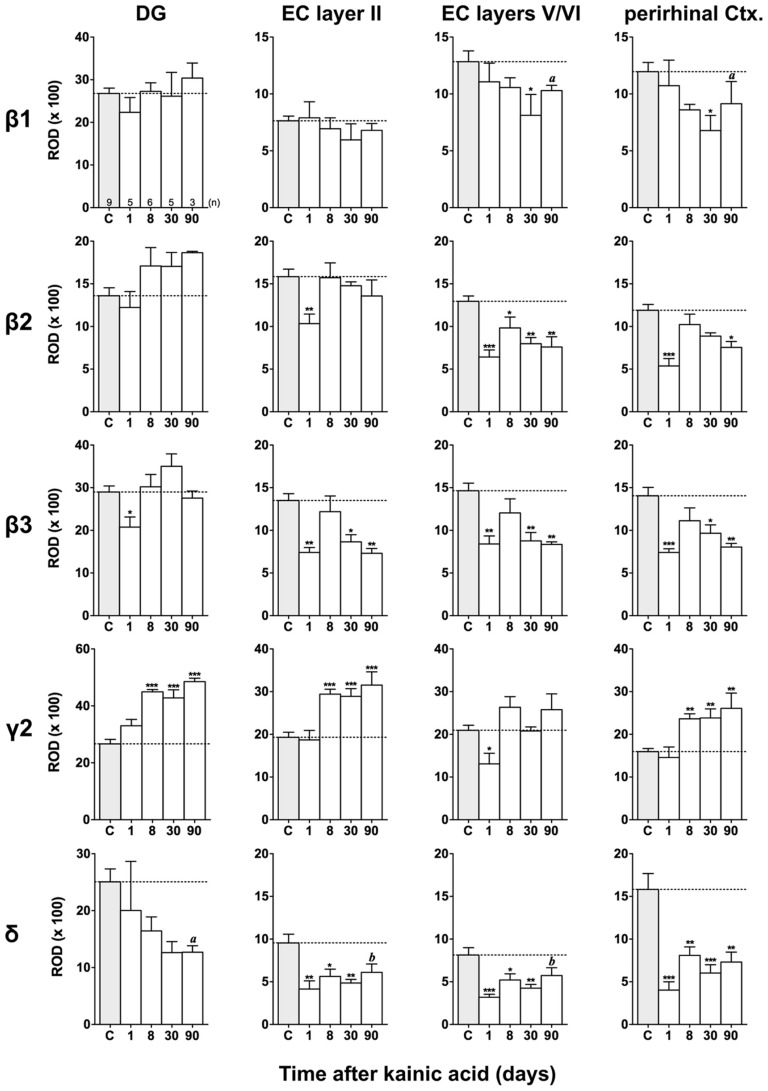
**Semi-quantitative assessment of mRNA levels for GABA_**A**_ receptor subunits β1–β3, γ2, and δ in the dentate gyrus (DG), entorhinal cortex (EC), and perirhinal cortex (PRC) after KA-induced seizures.** Note the reduced β subunit mRNA levels in the superficial (β3) and deep entorhinal cortex (EC; β1–β3) and in the PRC (β1–β3) at late intervals after KA. While subunit δ mRNA levels were decreased in the DG, entorhinal, and PRC at all intervals after KA, γ2 mRNA levels were increased in the DG, superficial EC, and PRC at late intervals. Numbers of rats per group are given in the upper left graph. Data are shown as mean ROD values ± SEM; statistical analysis was done by ANOVA and Dunnett’s multiple comparison *post hoc* test (*p < 0.05; **p < 0.01; ***p < 0.001). In cases where, due to the low number of animals, no significant difference was found for the 30 and 90 days intervals, data for this time points were pooled and re-analyzed: *a*, *p* < 0.05; *b*, *p* < 0.01.

Film autoradiographs after *in situ* hybridization are shown in **Figures 2** and **3**. ROD values obtained by densitometrical analysis of transcript levels are depicted in **Figures 4–7**. In addition to the brain areas depicted we also examined separately layers II and V/VI of the medial and lateral EC. There was no significant difference in the expression level of GABA_A_ receptor subunits between the medial and lateral parts of the EC. We therefore pooled the data obtained in the medial and lateral EC and depict them as “EC layer II” and “EC layers V/VI.”

#### Changes in α1 and α5 mRNAs after KA-induced seizures

Expression of α1 mRNA was significantly decreased in sector CA3, proximal and distal subiculum, and EC (deep layers) 1 day after status epilepticus (**Figures 2, 4**, and **5**). At later intervals, however, α1 mRNA concentrations increased again in these regions and were similar to or exceeded expression levels in controls. Significantly increased levels of α1 mRNA were present 30 and 90 days after KA injection in the DG, sectors CA3 and CA1, and in the EC (layer II) and PRC (**Figures 4 and 5**).

Messenger RNA encoding for the α2-subunit was significantly reduced in the DG, hippocampus proper, subiculum, and PRC 1 day after KA injection. While α2-subunit mRNA concentrations in the DG, hippocampus proper, and PRC increased at later intervals (30 and 90 days) to levels observed in controls, α2 mRNA levels in the subiculum were still reduced by about 45–60% after 90 days (**Figures 4** and **5**). As shown in **Figure 5**, expression of α3 mRNA did not change in the granule cell layer of the DG over the course of the experiment. The other areas investigated revealed transiently decreased expression of α3 mRNA 1 and 8 days after KA injection. These decreases were compensated by approaching control levels in most parts of the hippocampal formation, however were markedly increased (220% of controls) in the sector CA1 after 90 days (**Figure 4**).

Subunit α4 mRNA concentration was reduced by 35–45% in the hippocampus proper 1 and 8 days after KA injection (**Figure 4**) but reached approximately control levels at later time intervals (30 and 90 days). Also in the proximal subiculum, in the EC (layer II), and in the PRC, α4 mRNA levels were decreased by about 30, 25, and 35%, respectively, after 24 h. At later intervals (30 and 90 days) α4 mRNA levels, however, increased in the DG (about 170% of controls) and in the proximal subiculum (about 130–170% of controls).

Subunit α5 mRNA showed the most drastic and widespread changes in its expression. Considerably decreased concentrations of α5 mRNA were already evident 1 day after KA injection in all investigated subregions and ranged from about -60% in the proximal subiculum to about -75% in the DG and hippocampal sector CA1 (**Figures 4** and **5**). After a transient increase in expression after 8 or 30 days, α5 mRNA was again decreased after 90 days (from -50 to -80%) in all investigated regions.

#### Changes in β1–β3 mRNAs after KA-induced seizures

**Figures 3, 6**, and **7** show changes in the expression of β-subunit mRNAs. In the hippocampus proper, mRNA expression for the β1-subunit was significantly reduced from 1 to 30 days after KA injection, but almost reached control levels after 90 days (**Figure 6**). In the subiculum, β1 mRNA expression was permanently decreased by about 30–40%. The expression of β1 mRNA was unchanged in the granule cell layer of the DG and in layer II of the EC and was only transiently down-regulated after 30 days in the deep layers of the EC and in the PRC (**Figure 7**). KA-induced changes in the expression of subunits β2 and β3 were almost identical. As shown in **Figures 6** and **7**, both subunit mRNAs were significantly down-regulated (by up to 65%) in virtually all investigated regions (except β2 mRNA in the DG and sector CA1) 24 h after KA injection. While expression levels of subunit β2 later recovered close to control levels in the hippocampus proper and in layer II of the EC or even exceeded control levels (DG), its expression in the remaining areas only transiently recovered after 8 days but decreased again after 90 days (by about 40–50%). Similarly, expression of subunit β3 recovered after 8–30 days but declined again by about 25– 50% after 90 days in all regions but the DG (**Figures 3, 6, and 7**).

#### Changes in γ2 and δ mRNAs after KA-induced seizures

Expression of γ2 mRNA was transiently decreased by 40–60% in sectors CA3 and CA1 of the hippocampus, in the proximal subiculum, and in deep layers of the EC 1 day after KA injection (**Figures 6** and **7**). At later time intervals, we observed significantly increased expression of γ2 mRNA in the granule cell layer of the DG (up to 180% of controls), in layer II of the EC (up to 165%), and in the PRC (up to 165%). In the remaining regions, there was a (statistically not significant) trend for increased γ2 mRNA levels at the 30 and 90 days intervals. Expression of mRNA encoding the δ-subunit was lastingly decreased in all regions except sector CA3 and at all time points investigated (**Figures 6** and **7**). Ninety days after the initial status epilepticus, we observed about 50% decreased subunit δ mRNA levels in the DG, in the sector CA1 and in the PRC. In the other hippocampal and parahippocampal areas, subunit δ mRNA expression was reduced by 10–30%.

## DISCUSSION

We now report changes in the mRNA expression of 10 GABA_A_ receptor subunits in the hippocampal formation and in parahippocampal regions between one and 90 days after KA-induced status epilepticus. The main findings are (1) transient decreases in mRNA levels of all α-subunits, in subunits β2 and β3 and of subunit γ2 mRNA in the proximal subiculum and in the EC layer V/VI 24 h after KA injection, (2) lastingly decreased expression of subunits α5 and δ (with an onset at day 1 after KA injection) virtually in all hippocampal and parahippocampal areas (for subunit δ most prominently seen in the DG and the PRC, and for subunit α5 in sectors CA1 to CA3, the subiculum and the ento- and perirhinal corties where these subunits are most prominently expressed in controls), (3) increased expression of α4-subunit mRNA in the DG and in the proximal subiculum (30 and 90 days after KA), (4) increased expression of γ2-subunit mRNA in the DG, sector CA1, layer II of the EC, and PRC at late intervals after KA injection (30–90 days after KA), (5) in contrast, we observed lastingly decreased levels of α2- and of all β-subunit mRNAs in the subiculum and of β2- and β3-subunit mRNAs in the perirhinal and deep entorhinal cortices, (6) and increased expression of subunit α1 mRNA in the DG, hippocampus proper, superficial EC, and PRC 30 and 90 days after KA.

Our data reflect semi-quantitatively assessed mRNA levels. They likely reflect respective changes in the mRNA expression, which are mostly translated into protein (Schwarzer et al., 1997; Tsunashima et al., 1997; Nishimura et al., 2005). It has also always to be considered that neurodegeneration may obscure the results of mRNA expression. Neurodegeneration was most severe in the CA1 and CA3 sectors of the hippocampus, in parts of the subiculum and in layers II/III of the EC. And, neurodegeneration was almost maximal already after 24 h (Drexel et al., 2012), the earliest time interval reported here. In brain areas undergoing significant neurodegeneration, decreased mRNA levels may be due to this pathological change and increased mRNA concentrations could be apparently reduced by the underlying cell losses. Therefore it is always advisable to view the time course of changes and to compare changes in different subunits in the same brain area and of one subunit in different brain areas.

Decreases in subunit α1- and γ2-immunoreactivities due to rapid internalization were reported during the status epilepticus (induced by KA, pilocarpine or electrically; Brooks-Kayal et al., 1998; Naylor and Wasterlain, 2005; Nishimura et al., 2005). Since the α-subunits are crucial for the binding of benzodiazepines, it was suggested that this event may be causatively related to the partial resistance to benzodiazepine treatment during status epilepticus (Brooks-Kayal et al., 1998; Naylor and Wasterlain, 2005). Also our present study demonstrates an initial decrease in mRNA expression of these subunits in several hippocampal areas. This indicates that the reported internalization of α1- and γ2-subunits is accompanied by decreased expression of these subunits. These initial decreases in mRNA level, however, were followed by rapid overexpression of subunit γ2 mRNA and protein in all subfields of the hippocampus and may compensate for the initial losses (Schwarzer et al., 1997; Nishimura et al., 2005). Interestingly, our present study also revealed that mRNA levels of almost all other subunits transiently decreased in their expression. Thus, the transient down-regulation of the GABA_A_ receptor subunits may be more general and may affect a great number of differently assembled receptors.

### CHANGES IN SUBUNITS MEDIATING TONIC INHIBITION

Inhibition via GABA_A_ receptors comprises phasic inhibition by activating GABA_A_ receptors at the synapse and tonic inhibition by stimulating high affinity GABA_A_ receptors located at peri- and extrasynaptic sites (Mohler et al., 1996; Semyanov et al., 2004; Farrant and Nusser, 2005). Tonic inhibition is responsible for about 75% of the total inhibitory charge received by hippocampal principal neurons (Mody and Pearce, 2004). Receptors containing the γ2-subunit are mainly located within the synaptic cleft and thus primarily are involved in generation of phasic inhibition. Key components of GABA_A_ receptors implicated in tonic inhibition in the DG and hippocampus proper are subunits α5, α4, and δ (Nusser et al., 1998; Caraiscos et al., 2004). Additionally, subunit α4 is considered to be the main partner of the δ-subunit in the thalamus and forebrain (Sur et al., 1999). Epilepsy-induced decreased expression of GABA_A_ receptor subunits δ and α5 notably in the DG and sectors CA1 and CA3, respectively, were observed in different animal models of epilepsy including the KA model, mouse and rat models of pilocarpine injection, kindling, and electrically induced status epilepticus (Schwarzer et al., 1997; Tsunashima et al., 1997; Fritschy et al., 1999; Houser and Esclapez, 2003; Peng et al., 2004; Nishimura et al., 2005). Here, we report that epilepsy-induced reduction of α5- and δ-subunit mRNA expression is not restricted to the DG and hippocampus proper, but is also present in the subiculum and in the entorhinal and perirhinal cortices.

Down-regulation of GABA_A_ receptor subunits that usually mediate tonic inhibition under control conditions may result in weakened or diminished tonic inhibition in the particular region. Surprisingly, however, GABA-mediated tonic inhibition in the hippocampus and DG seems to be preserved or even increased despite the reduced expression of the respective GABA_A_ receptor subunits (Scimemi et al., 2005; Zhang et al., 2007; Zhan and Nadler, 2009; Rajasekaran et al., 2010). The cause for the apparent lack of effect of decreased expression of subunits that mediate tonic inhibition (α5 and δ) is not yet clear. A possible explanation may be a change in the composition of GABA_A_ receptors mediating tonic inhibition. This may also include a translocation of γ2-subunit containing receptors typically found within the synapse to extra- or perisynaptic sites, or the formation (up-regulation) of extrasynaptic receptors containing only α- and β-subunits, or a compensatory up-regulation of α4 subunits assembling to α4βγ2 receptors (Mortensen and Smart, 2006; Zhang et al., 2007). Here we found neurochemical evidence for a substitution in the expression of subunit δ by γ2, and for a (almost general) loss in subunit α5 and a (restricted) gain in subunit α4.

In conclusion, our data demonstrate considerable changes in the expression of most GABA_A_ receptor subunits in parahippocampal areas including the subiculum, the EC and the PRC. These changes are often consistent with those observed in the DG and hippocampus proper. Notably subunits α5 and δ are down-regulated in most areas, whereas up-regulation was observed for subunits γ2 and α4.

## Conflict of Interest Statement

The authors declare that the research was conducted in the absence of any commercial or financial relationships that could be construed as a potential conflict of interest.
